# Urine Proteomic Study in OAB Patients—Preliminary Report

**DOI:** 10.3390/jcm9051389

**Published:** 2020-05-08

**Authors:** Konrad Futyma, Łukasz Nowakowski, Alicja Ziętek-Strobl, Aleksandra Kamińska, Nadia Taoussi, Tomasz Rechberger

**Affiliations:** 1 2nd Department of Gynecology, Medical University of Lublin, Jaczewskiego 8, 20-954 Lublin, Poland; luknowakow@gmail.com (Ł.N.); zietek.alicja@yahoo.com (A.Z.-S.); aleksandra_filipczak@op.pl (A.K.); rechbergt@yahoo.com (T.R.); 2 Accident and Emergency Department, Haukeland University Hospital, N-5021 Bergen, Norway; nad.taou@gmail.com

**Keywords:** overactive bladder, proteomics, vascular cell adhesion molecule-1, urgency urinary incontinence, lower urinary tract symptoms

## Abstract

Overactive bladder (OAB) is defined by International Urogynecological Association (IUGA)/ International Continence Society (ICS) as urinary urgency, usually accompanied by frequency and nocturia, with or without urgency urinary incontinence, in the absence of urinary tract infection (UTI) or other obvious pathology. The pathophysiology of OAB is not well understood, however a number of different proteins and cytokines including vascular cell adhesion molecule-1 (VCAM-1) were found to be important in regulating structural integrity of the bladder wall. Proteome analysis may thus provide significant information with regard to OAB and may help in discovering novel diagnostic disease biomarkers. Sixteen Caucasian women aged 32–78 were included in the study. Patients were placed within 2 groups: OAB group (*n* = 8) and control group (*n* = 8). Urine samples were collected, immediately preserved in a protease inhibitor mixture, and frozen at −80 ℃. All samples were then further processed according to the isobaric tags for relative and absolute quantification (iTRAQ) manual. Proteins were labeled and analyzed in the mass spectrometer conjugated with liquid chromatograph (data are available via ProteomeXchange with the identifier PXD017799). There were no statistically significant differences in demographic data between control and OAB groups. VCAM-1 was the only protein that reached statistical significance as a differentiating protein in both of our experiments assessing the proteomic constitution in OAB patients. Studies involving a larger group of patients may provide further information on urinary bladder proteomics.

## 1. Introduction

Overactive bladder syndrome (OAB) is defined by International Urogynecological Association (IUGA)/ International Continence Society (ICS) (IUGA/ICS) as a form of urinary urgency, usually accompanied by frequency and nocturia, with or without urgency urinary incontinence, in the absence of urinary tract infection (UTI) or other obvious pathology [1]. There are several theories regarding the pathophysiology of OAB and detrusor overactivity (DO) including nervous, myogenic, and urothelial involvement but a full explanation is still missing. However, recent genetic studies have shed a light on gene expression engagement on OAB onset. Accordingly, several genes were found to be either over- or under-expressed in OAB sufferers. The use of micro- and macro-array as a methods of elucidating gene changes involved in the development of urgency was crucial for detecting genetic changes associated with this condition. Platelet-derived growth factor (PDGF), microfibrillar-associated protein (MAP), vascular cell adhesion molecule-1 (VCAM-1), and tropomyosin were found to be important in regulating the structural integrity of the bladder and its supporting tissues [2,3]. However, due to post-translational changes, encoded proteins may be altered and their impact on certain conditions can be limited. Proteome analysis may therefore provide significant pathophysiological information and may help in the discovery of novel diagnostic disease biomarkers or even in establishing new therapeutic methods [4]. Urine, being noninvasively collected, seems to be an ideal material for testing in patients with lower urinary tract symptoms (LUTS). Nevertheless, before urine proteomic markers become clinically known and useful, the urine proteome itself must be thoroughly characterized in diverse populations. In 2015, the National Institute of Diabetes and Digestive and Kidney Disease (NIDDK) invited experts of different specialties to a Urinology Think Tank meeting, and they concluded that features, such as the chemical and physical characteristics of urine, including the microbiota, cells, pH, metabolites, and proteins, might interact in complex ways with one another and with the bladder and/or kidney to potentially affect overall health [5]. Recently, Mossa et al. found that urinary metabolites might be involved in the pathophysiology of OAB and can be helpful in its diagnosis. In their work, increased urinary levels of mitochondrial dysfunction markers, oxidative stress, and ketosis correlated significantly with the OAB symptoms scores obtained through questionnaires. Moreover, multiple linear regression modeling revealed that age, blood glucose, and urine metabolites (malate, fumarate, and α-hydroxyisobutyrate) were significant predictive factors of OAB severity [6].

Other work has shown that the urothelium is responsible not only for isolating the bladder environment from surrounding tissues, but it also plays a crucial role in molecular transport through the bladder wall via numerous functional receptors, junctions, and channels (i.e., aquaporins, ion channels, gap junctions) [7]. Growth factors, cytokines, or proteins such as the epithelial growth factor (EGF); fibroblast growth factor receptor (FGFR), nerve growth factor (NGF), brain-derived neurotrophic factor (BDNF), vascular cell adhesion molecule (VCAM-1), and inter-cellular adhesion molecule 1 (ICAM-1) were found to alter urothelial functions in specific circumstances [3,8,9].

The aim of this study was to analyze the urinary proteomic pattern in patients suffering from OAB symptoms and to establish a quantitative and qualitative protein profile for OAB that may be supportive in defining the mechanism of the OAB pathophysiology.

## 2. Materials and Methods

Sixteen Caucasian women aged 32–78 were included within the study. Prior to the study, all patients signed informed consent and agreed to the use of the obtained data for scientific purposes. Moreover, data acquisition and analysis were performed in compliance with protocols approved by the Medical University of Lublin Ethical Committee (ethical approval number KE-0254/46/2016). In the study, participants were placed within 2 groups: OAB (*n* = 8) and control (*n* = 8). Inclusion criteria for patients with OAB symptoms were as follows: average of ≥8 micturitions/day during a 3 day bladder diary period and at least 1 episode of urge urinary incontinence (UUI) a day. Patients from the OAB group did not have any other comorbidities (except OAB) and did not take any medications prior to the study. Patients from the control group just had a routine gynecological check-up and were free of any LUTS and any other comorbidities as well and did not take any medications prior to the study. Urine samples were (40 mL) collected using a 14 Fr sterile catheter, immediately preserved in a bacteriostatic factor (sodium azide) and Pefabloc, protease inhibitor mixture (Sigma-Aldrich, Saint Louis, MI, USA), and frozen at −80 ℃. The obtained urinary proteins were precipitated by applying the Wessel–Fluegge method [10], and all samples were then further processed according to the the isobaric tags for relative and absolute quantification (iTRAQ) manual (Applied Biosystems, Foster City, CA, USA). Forty micrograms of the proteins was taken from each sample and digested with trypsin at 37 °C (Promega, Madison, WI, USA). Samples were than labeled and analyzed in the Q Exactive™ Hybrid Quadrupole-Orbitrap™ Mass Spectrometer (MS) (Thermo Fisher Scientific, Waltham, MA, USA) conjugated with the high-efficiency nanoACQUITY UPLC^®^ liquid chromatograph (LC) (Waters Corporation, Milford, MA, USA). Detailed sample processing is described elsewhere [11] and a separate Supplementary File (File S1) has been submitted. Samples were analyzed in duplicate. Samples were divided into two separate experiments with 4 OABs and 4 controls in each described as ExpOABK_01 and ExpOABK_02. Acquired data were analyzed using the MASCOT engine (Matrix Science, London, UK) to search the SWISS-PROT protein database, limited to the *Homo sapiens* taxonomy. After the data arrangement, it was put through a second search using the DECOY database. Statistical analysis was performed by way of the Diffprot software, a resampling-based software tool for statistical analysis of data derived from MS-based proteomic experiments [12]. For demographic analysis Statistica 12.0 PL (StatSoft Polska Sp. z o. o., Krakow, Poland) and the chi^2^ test were used. “Ratio” is the parameter that says whether the protein is under- or over-expressed. A value of <1.0 means that this protein is under-expressed in OAB patients, and a value >1.0 means it is over-expressed. Fold change describes how great the difference is, no matter the direction. The mass spectrometry proteomics data have been deposited in the ProteomeXchange Consortium via the PRIDE [13] partner repository with the dataset identifier PXD017799 and 10.6019/PXD017799. Reviewer account details: Username: reviewer58080@ebi.ac.uk; Password: hPZXyBVu.

## 3. Results

There were no statistically significant differences in demographic data between control and OAB groups (Table 1).

**Figure 1:**
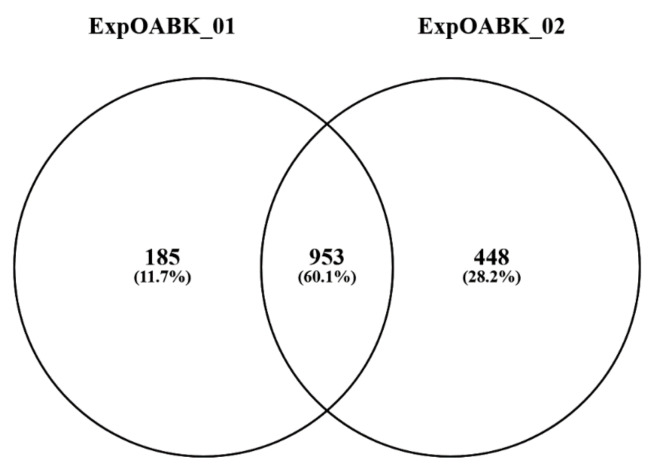
Results of qualitative analysis—a Venn diagram representing the number of proteins identified by two or more peptides in two biological replicates of the iTRAQ experiment. Here, 953 proteins are common to both experiments. iTRAQ, isobaric tags for relative and absolute quantification.

The proteome of urine collected from 8 OAB patients and 8 healthy subjects was compared using a combined liquid chromatography–mass spectrometry (LC-MS) relative quantitation of iTRAQ labeled peptides. The sample labeling yield rate with iTRAQ markers for both experiments was 99.7%. From each sample, an equal amount of total protein obtained in urine retentate, after filtration on >10 kDa cutoff filters, was used for the analysis. Qualitative results (protein lists) from two LC-MS experiments were combined, resulting in a dataset with all 953 proteins identified by at least two peptides (Figure 1). Within this dataset, protein identifications based on identical peptide sets were again grouped and each group was treated as a single protein cluster in further processing.

Qualitative proteins, that significantly differed between the OAB and control group in both experiments are presented in Table 2. Herein, vascular cell adhesion molecule-1 was the only protein found to have a *q* value <0.05 in both experiments. In addition, ratio and fold change were similar in both experiments. The complete list of differentiated proteins and full data is provided in separate Supplementary Files: first experiment: Full list ExpOABK_01.xlsx and second experiment: Full list ExpOABK_02.xlsx (Table S1 and Table S2, respectively). All identifications in both experiments are given in Table 3.

## 4. Discussion

There are several theories concerning the pathophysiology of OAB and UUI, including that it comes about urothelial changes (deteriorating urine–blood barrier function), myogenic alterations (bladder smooth muscle contractility and excitability), and neurogenic dysfunctions (increased or sensitized sensory mechanisms in the central nervous system) [14,15]. In the gastrointestinal tract, interstitial cells of Cajal (ICCs) act as pacemakers, driving peristaltic activity throughout the gut and also have a key role in the transmission of signals from nerves to smooth muscle. What is more, they were found to be able to mediate cholinergic signaling in the urinary bladder, thus, supporting the existence of a sensory transduction signaling system between urothelium, sensory neurons, and the underlying detrusor muscle fibers [16,17]. Thus, increased ICC number, along with gap-junction density in the bladder wall might be responsible for OAB symptoms occurrence [18,19]. Furthermore, a number of urinary inflammatory biomarkers have been found to be elevated in OAB patients, as well as in bladder pain syndrome/interstitial cystitis (BPS/IC), including monocyte chemotactic protein-1 (MCP-1), macrophage inflammatory protein (MIP-1β), tumor necrosis factor-α (TNF-α), granulocyte colony-stimulating factor (GC-SF), and epidermal growth factor receptor (EGFR) or eotaxin. In addition, altered synthesis of several proteoglycans, cell adhesion, and tight junction proteins might predispose to frequency and urgency symptoms manifestation [20]. Beyond the aforementioned, monocyte chemotactic protein-1 provokes mast cell activation and has chemotactic activity for monocytes. Moreover, MIP-1β released from mast cells and macrophages can bind heparin and has inflammatory and neutrophil chemokinetic properties, whereas eotaxin possesses selective chemotactic activity for eosinophils. Furthermore, chemokines activate target cells in the bladder, and thereby contribute to the inflammatory-induced changes in the bladder wall tissues [21,22]. In our work, vascular cell adhesion molecule-1 was the only protein that reached statistical significance, as a differentiating protein, in both experiments when assessing the proteomic constitution in OAB patients. Interestingly, this is consistent with the results published by Corcoran et al., where VCAM-1 and ICAM-1 were found to be the most possibly indicative cytokines for interstitial cystitis/painful bladder syndrome (IC/PBS) [3]. Overactive bladder syndrome and IC/PBS are considered to be separate pathological conditions, however, there is now significant scientific evidence that both are related to structural, synaptic, and complex signaling pathway changes that triggers altered bladder sensation [23,24].

Vascular cell adhesion molecule-1, a member of chemo-attractant cytokines family, is a protein that mediates the adhesion of the inflammatory cells to the endothelium and is responsible for the leukocyte–endothelial cell signal transduction. Thus, it may play an important role in the urothelium pathology involving changes in cell-to-cell integrity and the damaging of urothelial barrier function due to inflammatory processes [25].

In various animal model experiments, VCAM-1 blockade reduces the severity of inflammatory reaction in atopic dermatitis, multiple sclerosis, and Crohn’s disease, mostly by blocking T cell infiltration into the affected tissues [25,26]. Injection of anti-VCAM-1, blocking antibodies also inhibits eosinophil recruitment in asthma models in several species [27,28].

The limitations of our preliminary study are the small number of patients in each group, that only females were included, and that only duplicate analysis was done for each sample. Other issues include the absence of an orthogonal method for the validation of the significantly different levels of VCAM-1 between the two groups and a lack of parallel serum proteome analysis in order to ascertain whether the protein differences are found only in the urine. Studies on a larger group of patients accompanied by other parallel testing methods may provide further information on urinary bladder proteomics—which potentially might be supportive for novel diagnosis and treatment options.

## 5. Conclusions

Our working theory is that OAB might be one of several allergic or immunogenic conditions that involve inflammatory reactions driven by VCAM-1 and other proinflammatory cytokines. Preliminary results of this OAB proteomics study indicated that there are differences of proteome between OAB patients and healthy subjects that might suggest that autoimmune response is, at least partially involved in OAB onset.

## Figures and Tables

**Table 1:** Demographic data of the study groups.

Parameter	OAB Group (*n* = 8)	Control Group (*n* = 8)	*p* Value
Age (years)	57.4	62.0	0.08
BMI (kg/m^2^)	24.1	25.2	0.69
Parity (*n*)	1.9	1.5	0.44

OAB, overactive bladder; BMI, body mass index.

**Table 2:** Qualitative and quantitative results of the differential proteins in OAB vs. control group in both experiments (ExpOABK_01 and ExpOABK_02).

**Experiment 1 (ExpOABK_01)**						
**No.**	**Protein**	***q* Value**	**Ratio**	**Fold Change**	**Peptides**	**Description**
1	P25311	0.0002	1.46	1.46	40	Zinc-alpha-2-glycoprotein
2	P12830	0.0002	1.45	1.45	28	Cadherin-1
3	P06396	0.0002	1.37	1.37	30	Gelsolin
4	P02760	0.0009	1.15	1.15	50	Protein AMBP
5	P41222	0.00391	1.21	1.21	53	Prostaglandin-H2 D-isomerase
6	P08246	0.00397	0.43	2.35	4	Neutrophil elastase
7	Q9Y279	0.00465	2.17	2.17	4	V-set and immunoglobulin domain-containing protein 4
8	**P19320**	**0.00723**	**1.36**	**1.36**	**10**	**Vascular cell adhesion molecule 1**
9	P0DOX8	0.06346	1.1	1.1	32	Immunoglobulin lambda-1 light chain
10	Q5T013	0.02613	1.25	1.25	18	Putative hydroxypyruvate isomerase
11	P01619	0.02754	1.19	1.19	9	Immunoglobulin kappa variable 3–20
**Experiment 2 (ExpOABK_02)**						
**No.**	**Protein**	***q* Value**	**Ratio**	**Fold Change**	**Peptides**	**Description**
1	P98160	0.00018	0.76	1.32	60	Basement membrane-specific heparan sulfate proteoglycan core protein
2	Q9HCU0	0.00018	0.49	2.05	5	Endosialin
3	P02787	0.00018	1.4	1.4	47	Serotransferrin
4	P00738	0.00018	2.07	2.07	13	Haptoglobin
5	P20742	0.00029	1.36	1.36	43	Pregnancy zone protein
6	P0DOX7	0.00172	1.23	1.23	34	Immunoglobulin kappa light chain
7	P06870	0.00652	0.53	1.87	7	Kallikrein-1
8	P05155	0.00704	0.75	1.34	38	Plasma protease C1 inhibitor
9	P04083	0.00735	1.53	1.53	15	Annexin A1
10	O95460	0.01472	0.47	2.11	3	Matrilin-4
11	P02750	0.01886	1.37	1.37	25	Leucine-rich alpha-2-glycoprotein
12	P36957	0.01934	0.34	2.97	2	Dihydrolipoyllysine-residue succinyltransferase component of 2-oxoglutarate dehydrogenase complex. mitochondrial
13	P13647	0.01991	0.56	1.78	7	Keratin. type II cytoskeletal 5
14	P0DOX5	0.01992	1.33	1.33	11	Immunoglobulin gamma-1 heavy chain
15	**P19320**	**0.02104**	**1.24**	**1.24**	**12**	**Vascular cell adhesion molecule 1**
16	P01861	0.02134	1.47	1.47	9	Immunoglobulin heavy constant gamma 4
17	Q99715	0.03727	0.8	1.26	27	Collagen alpha-1(XII) chain
18	P22105	0.03768	0.78	1.28	38	Tenascin-X

The blod shows that only this protein was significantly changed in both experiments as mentioned in the text.

**Table 3:** The number of identified peptides and proteins in both experiments.

Sample	Total Queries	Queries	Peptides	Proteins
ExpOABK_01	1,388,534	19,605	5529	1138
ExpOABK_02	1,434,226	22,496	7003	1401

## Supplementary Materials

The following are available online at https://www.mdpi.com/2077-0383/9/5/1389/s1, Table S1: Full list ExpOABK_01, Table S2: Full list ExpOABK_02, File S1: Detailed material and methods.
