# Supplementary material for: Urine Proteomic Study in OAB Patients—Preliminary Report
Source: J Clin Med. 2020 May 8;9(5):1389. doi: 10.3390/jcm9051389 (PMC7290998; doi:10.3390/jcm9051389)
Supplement: Supplementary file 1 [file jcm-09-01389-s001.zip › jcm-764005-supplementary-for conversion/File S1 Detailed material and methods.docx]

**Quantitative Analysis of the Protein Fraction Obtained from Human Urine**

1. Handling of the Biological Material

1. Samples were stored at −80 °C prior the experiment.
2. Thawed material was mixed and centrifuged (10‘, 3000 rcf).
3. Supernatants were filtered through 0.45 µm filters, pellets were kept frozen at −80 °C.

**Characteristics of the samples:**

| **Name** | **Sediment Amount** | **Supernatant** | |
| --- | --- | --- | --- |
|  |  | **pH** | **Protein Concenrtation (µg/µL)** |
| 1 OAB | + | 7 | 0.03 |
| 2 OAB | +++ | 5.5 | 0.01 |
| 3 OAB | ++ | 6 | 0.01 |
| 4 OAB | - | 7 | 0.1 |
| 5 OAB | +++ | 7 | 0.01 |
| 6 OAB | - | 7 | 0.07 |
| 7 OAB | +++ | 5.5 | 0.06 |
| 8 OAB | ++ | 5.5 | 0.01 |
| 9 K | + | 6 | 0.01 |
| 10 K | + | 6 | 0.02 |
| 11 K | + | 6.5 | 0.01 |
| 12 K | +++ | 6 | 0.04 |
| 13 K | ++++ | 5 | 0.03 |
| 14 K | +++ | 5 | 0.03 |
| 15 K | ++++ | 6.5 | 0.04 |
| 16 K | + | 5.5 | 0.01 |

OAB, overactive bladder; +, ++, +++, ++++, visual assessment of sediment amount.

Due to the low protein concentration in the samples, the material was concentrated on Speed-vac.

2. Protein Fraction Isolation

1. Due to the presence of protease inhibitors added to the samples by the researchers, it was necessary to precipitate the proteins from the solution. The protein fraction was obtained by Wessel-Fluegge [Reference: Wessel, D. Fluegge, U.I. (1984), Anal. Biochem. 138, 141–143].
2. Precipitated urine sediments were dissolved according to the manufacturer’s instructions for the isobaric tags for relative and absolute quantification (iTRAQ) protocol (Applied Biosystems). Protein concentration was measured.

| **Name** | **Protein Concentration (µg/µL)** | **Protein Quantity (µg)** |
| --- | --- | --- |
| 1 OAB | 1.24 | 49 |
| 2 OAB | 3.61 | 144 |
| 3 OAB | 1.69 | 67 |
| 4 OAB | 1.02 | 40 |
| 5 OAB | 5.66 | 226 |
| 6 OAB | 2.29 | 91 |
| 7 OAB | 5.03 | 201 |
| 8 OAB | 2.47 | 197 |
| 9 K | 2 | 200 |
| 10 K | 4.68 | 468 |
| 11 K | 1.69 | 67 |
| 12 K | 2.82 | 282 |
| 13 K | 2.27 | 272 |
| 14 K | 1.9 | 190 |
| 15 K | 1.91 | 191 |
| 16 K | 2.4 | 288 |

3. Protein Digestion and Peptide Labeling

1.40. µg of proteins was taken from each sample.

1. Proteins were reduced by addition of 4 mL Tris (2-carboxyethyl) phosphine hydrochloride solution (TCEP) to each sample, incubation for 1 hr at 60 ℃.
2. Reduced cysteines were blocked by addition of 2 mL of methyl methanethiosulfonate (MMTS), incubation for 10′ at room temperature.
3. Proteins were digested with trypsin (Promega, Madison, WI, USA) in a 1:20 ratio, incubation at 37 °C with shaking.
4. Samples were labelled according to the scheme listed below and in accordance with the manufacturer’s protocol:

| **ExpOABK 01:** | | **ExpOABK 02:** |
| --- | --- | --- |
| 113 | -9 K | 113-13 K |
| 114 | -10 K | 114-14 K |
| 115 | -11 K | 115-15 K |
| 116 | -12 K | 116-16 K |
| 117 | -1 OAB | 117-5 OAB |
| 118 | -2 OAB | 118-6 OAB |
| 119 | -3 OAB | 119-7 OAB |
| 121 | -4 OAB | 121-OAB |

6 Samples with labelled peptides were combined according to the scheme and concentrated on a Speed-vac.

4. Clean-Up of Labelled Samples, Lc-Ms / Ms Fractionation and Measurement

1. Two batches of mixed samples, ExpOABK 01 and ExpOABK 02, were purified on an Oasis columns and lyophilized.
2. Labelled peptides were separated by High pH fractionation (HpH) chromatography into 19 fractions.

Set-up: Waters XBridge BEH C_18_ Column, 130Å, 3.5 µm, 4.6 mm × 150 mm, 1/pkg [186003034]; Method: 21 min of acetonitrile gradient (2%–90%), (isocratic 20% Amonium Water pH 10); Each fraction was lyophilized, resuspended in 50 µl of 0.1 % TFA and measured on a QExactive (Thermo Fisher Scientific, Waltham, MA, USA) spectrometer coupled with a high performance liquid chromatograph (nanoACQUITY UPLC). Set-up: RP-C18 column 75 µm diameter, 250 mm long (Waters Corporation, Milford, MA, US), gradient: 160 min of acetonitrile gradient (5–35 % ACN) in the presence of 0.1 % formic acid. QExactive (Thermo Fisher Scientific, Waltham, MA, USA) dynamically switching between mass spectrometry MS and MS / MS measurement. Higher energy collisional dissociation fragmentation (HCD) was used to fragment the peptides.

5. Data Processing and Statistical Analysis \

1. The data obtained in the spectrometer was searched by a Mascot version 1.0 engine against the Swiss-Prot protein [SwissProt_2018_04 (557275 sequences] taxonomy was narrowed to Homo sapiens [Homo sapiens (human) (20416 sequences)]. Normalized data was searched again against the database, this time in conjunction with the decoy database to calculate the *q*-values for peptides.

- Enzyme – semiTrypsin
- Missed clevage – 1
- Fixed modification - Carbamidomethyl (C), iTRAQ8plex (K), iTRAQ8plex (N-term)
- Variable modification - Oxidation (M)
- Ion mass error tolerances: ±7.25 ppm (parent) and ± 0.018048 Da (fragment)

1. Peptides with a *q*-value ≤0.05 were selected for quantitative analysis.
2. Statistical analysis was performed with the Diffprot program [Reference: [J Proteomics.](https://www.ncbi.nlm.nih.gov/pubmed/22641154) 2012 Jul 16;75(13):4062–73].
